# Structural and Functional Dysregulation of the Brain Endothelium in HIV Infection and Substance Abuse

**DOI:** 10.3390/cells13171415

**Published:** 2024-08-24

**Authors:** Narendran Annadurai, Georgette D. Kanmogne

**Affiliations:** Department of Anesthesiology, College of Medicine, University of Nebraska Medical Center, Omaha, NE 68198-4455, USA; nannadurai@unmc.edu

**Keywords:** blood–brain barrier, HIV-associated neurocognitive disorder, endothelial cells, viral proteins, gp120, Tat, Nef, Vpr, cocaine, methamphetamine, alcohol, tobacco, morphine, cannabinoids

## Abstract

Blood–brain barrier (BBB) injury and dysfunction following infection with the human immunodeficiency virus (HIV) enables viral entry into the brain, infection of resident brain cells, neuronal injury and subsequent neurodegeneration leading to HIV-associated neurocognitive disorders (HAND). Although combination antiretroviral therapy has significantly reduced the incidence and prevalence of acquired immunodeficiency syndrome and increased the life expectancy of people living with HIV, the prevalence of HAND remains high. With aging of people living with HIV associated with increased comorbidities, the prevalence of HIV-related central nervous system (CNS) complications is expected to remain high. Considering the principal role of the brain endothelium in HIV infection of the CNS and HAND, the purpose of this manuscript is to review the current literature on the pathobiology of the brain endothelium structural and functional dysregulation in HIV infection, including in the presence of HIV-1 and viral proteins (gp120, Tat, Nef, and Vpr). We summarize evidence from human and animal studies, in vitro studies, and associated mechanisms. We further summarize evidence of synergy or lack thereof between commonly abused substances (cocaine, methamphetamine, alcohol, tobacco, opioids, and cannabinoids) and HIV- or viral protein-induced BBB injury and dysfunction.

## 1. Introduction

The blood–brain barrier (BBB) is a semi-permeable membrane that separates circulating blood from brain tissues, regulates the transport of oxygen and essential nutrients into the central nervous system (CNS), protects the brain from harmful substances, and maintains brain homeostasis [1,2,3,4]. It is primarily composed of endothelial cells (ECs) lining the brain blood vessels, a basement membrane that encases the ECs on the abluminal side, pericytes, and astrocyte endfeet [2,3,4]. The molecular components of the BBB responsible for its protective and barrier functions include endothelial tight junction (TJ) proteins (occludin, claudins, and zonula occludens (ZO)), adherent junction (AJ) proteins, and efflux transporters [2,3]. Endothelial injury and subsequent BBB dysfunction are implicated in the pathogenesis of several CNS infections and diseases, including human immunodeficiency virus (HIV) CNS infection and neuroHIV.

**Figure 1:**
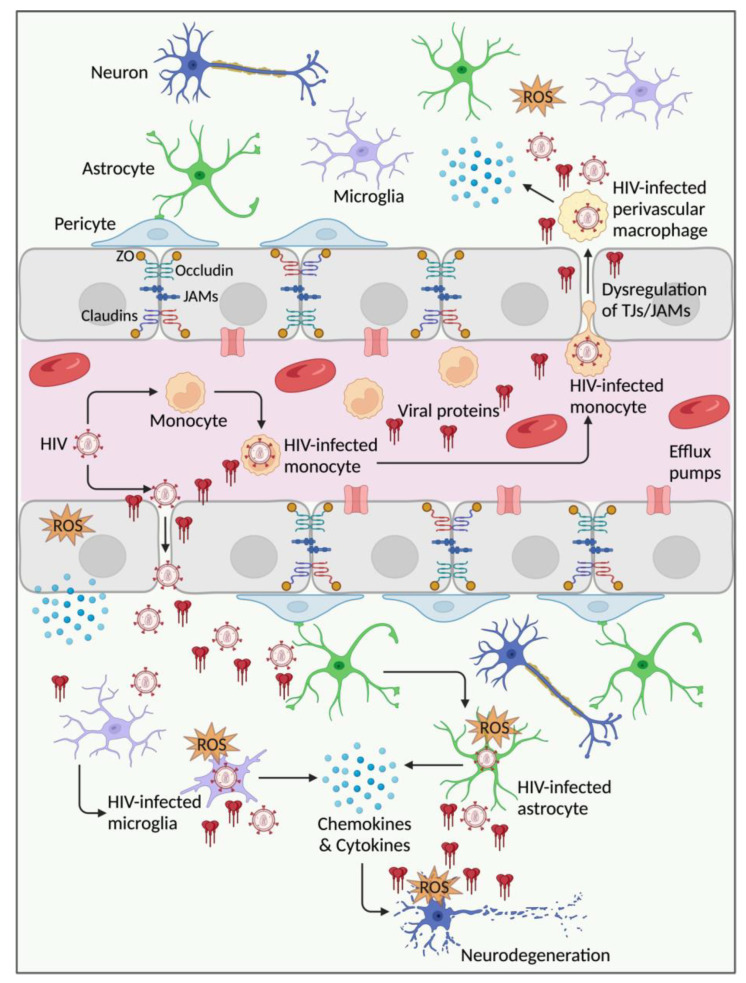
Schematic illustration of HAND neuropathogenesis. Following contact with the brain endothelium, circulating HIV-infected leukocytes, virions, and viral proteins (e.g., gp120, Tat, Nef) induce blood-brain barrier (BBB) injury, associated with increased inflammation and production of reactive oxygen species (ROS), damage to endothelial tight junction (TJ) proteins (occludin, claudins, zonula occludens), and damage to adherent junctions (junctional adhesion molecules (JAMs)) proteins. This results in BBB dysfunction, including increased permeability and infiltration of HIV and infected cells into the central nervous system (CNS). Once in the brain, HIV infects resident brain cells, including microglia (productive infection) and astrocytes. Productively infected brain cells further release HIV and viral proteins, inflammatory cytokines, chemokines, and ROS. These factors further increase BBB injury and dysfunction, CNS infection, and induce neuronal injury. Repeated and prolonged neuronal injury causes neurodegeneration, which can result in HIV-associated neurocognitive disorders (HAND). Created with BioRender.com (accessed on 14 June 2024).

In the early stages of HIV infection, endothelial injury following exposure to circulating infected leukocytes, virions, and viral proteins, as well as subsequent BBB dysfunction, enables HIV entry into the brain and infection of resident CNS cells, including productive microglial infection [5,6]. Virions and viral proteins released by infected brain cells are associated with increased inflammation and oxidative stress, which leads to neuronal injury and subsequent neurodegeneration [7,8] (Figure 1). Increased neurodegeneration over time results in motor, cognitive, and behavioral abnormalities termed HIV-associated neurocognitive disorders (HAND) [9,10]. HAND subclassification includes HIV-associated asymptomatic neurocognitive impairment, mild neurocognitive disorder, and HIV-associated dementia [10,11,12,13]. Although the incidence and prevalence of the most severe form of HAND has decreased with the advent of combination antiretroviral therapy (ART), the overall prevalence of HAND and its associated morbidity remains high (up to 40%) [14,15]. With increased life expectancy and aging of people living with HIV (PLWH) [16,17], the prevalence of HAND and HIV-associated CNS comorbidities is expected to remain high. Considering the principal role of the brain endothelium in HIV infection of the CNS and HAND, the purpose of this manuscript is to review the current literature on BBB structure and function in HIV infection and substance abuse. We summarize evidence from human and animal studies, in vitro studies, and associated mechanisms. We also summarize evidence of synergy, or lack thereof, between commonly abused substances (cocaine, methamphetamine, alcohol, tobacco, opioids, and cannabinoids) and HIV- or viral protein-induced BBB injury and dysfunction.

## 2. HIV Infection, BBB Injury, and Dysfunction

### 2.1. Evidence from Human Studies

Studies have shown that HIV-infected patients have BBB injury that is associated with increased permeability, infiltration of circulating leukocytes into the CNS, and advanced disease. In these studies, patients with HAND had significantly more damage to the brain endothelium, and increased BBB permeability was observed in 50% of AIDS patients compared to 22% in asymptomatic PLWH [18,19,20]. There is other evidence of increased BBB breakdown in HIV-infected humans. Contrast-enhanced magnetic resonance imaging of PLWH showed altered myoinositol/creatine (mI/Cr) in the basal ganglia associated with high plasma viral load [21]. High cerebrospinal fluid (CSF) albumin and CSF/serum albumin quotient (QAlb) in PLWH correlates with increased neuronal injury (higher CSF neurofilament-L) and impaired brain bioenergetics (lower N-acetylaspartate-to-creatine ratio) in the parietal gray matter [22]. 

Evidence suggests that ART does not prevent or abrogate HIV-induced BBB injury and can increase BBB permeability. In PLWH who had an abnormal QAlb and N-acetylaspartate-to-creatine ratio, one year of ART did not improve QA1b [22]. Impaired BBB integrity has been reported in both HIV-infected subjects with HAND and neuroasymptomatic PLWH, despite a decreased QA1b post-ART [17]. Although ART suppressed CSF and plasma viral loads, it did not significantly change albumin and neurofilament-L levels [19]. A study of virally suppressed patients with HAND showed BBB disruptions in the basal ganglia and frontal white matter, despite a lack of peripheral inflammation [23]. HIV-infected adults on suppressive ART also showed impaired BBB associated with increased levels of amyloid-β (1–42), and phosphorylated and total tau correlating with CSF albumin and QAlb [24]. 

Autopsy evidence of brain endothelium injury and impaired BBB in HIV-infected patients has included enlarged microvessels and loss of hippocampal capillaries associated with degeneration of perivascular tissues [25], fragmentation and loss of microvessels TJ proteins, including claudin-5, occludin, ZO-1 and ZO-2 in several brain regions (frontal cortex, basal ganglia, subcortical white matter, and cortical gray matter) [26,27,28]. Brain tissues of HIV-infected humans has also showed significantly more capillary loss and decreased TJs in patients with HAND compared to HIV-infected subjects without HAND and seronegative controls subjects [20,23,26,27,28,29]. 

### 2.2. Evidence from Animal Studies

*Primate models*: Simian immunodeficiency virus (SIV)-infected macaques, including animals with SIV encephalitis, showed endothelial activation and BBB injury, reduced expression of endothelial TJ proteins occludin and ZO-1, and fragmentation of TJs in the basal ganglia microvessels associated with increased pro-inflammatory cytokines and accumulation of perivascular macrophages in the CNS [30,31,32,33]. SIV-infected macaques showed increased expression of vascular cell adhesion molecule (VCAM)-1 that correlated with endothelial activation, BBB injury, and increased production of cytokines such as tumor necrosis factor (TNF)-α and interferon-γ [32,34]. SIV-infected animals also showed increased production of chemokines such as the chemokine ligand-5 (CCL5), macrophage inflammatory protein-1α and -1β, monocyte chemotactic protein-3, and interferon-inducible protein-10 [35]. These chemokines likely contribute to increased diapedesis and recruitment of circulating leukocytes into the brain. 

*Murine models*: Evidence from several HIV/AIDS murine models show that mice and rats infected with HIV-1 [36] or exposed to HIV-1 glycoprotein (gp)120 [37,38,39,40], transactivator of transcription (Tat) [41,42], or negative factor (Nef) [43,44] had reduced expression of cerebral TJ proteins (e.g., occludin, claudin-5, ZO-1 and ZO-2). These animals also had increased BBB disruption and permeability associated with upregulation of endothelial adhesion molecules, matrix metalloproteinases (MMPs), and reactive oxygen species (ROS) [37,38,39,40,41,42,43]. In HIV-infected humanized mice, treatments that prevented or reduced TJ downregulation and BBB dysfunction were also associated with less gliosis, reduced inflammation and leukocyte infiltration into the CNS, and less neuronal injury [36,45]. Induction of Tat expression in transgenic (Tg) mice resulted in BBB breakdown associated with increased leakage of circulating dyes into the brain, activation of perivascular macrophages, and microgliosis [42]. HIV-1 Tg26 mice and gp120 Tg mice showed reduced ZO-1 expression and increased BBB permeability, including enhanced leakage of albumin into the brain associated with increased intercellular adhesion molecule (ICAM)-1, VCAM-1, and substance-P [37,38]. Injection of HIV-1 gp120 proteins to rats induced ROS, caused the upregulation of MMP-2 and MMP-9, and reduced brain claudin-5 and laminin (major component of endothelial basement membrane) [39,40]. Injection of HIV-1 Nef protein into rats also induced BBB disruption, reduced claudin-5, and increased the permeability of the BBB and other vascular tissues. This was associated with increased MMP-9 activity, inflammation, and leukocyte infiltration into tissues [43,44]. MMP-9 inhibitors prevented Nef-mediated BBB injury [43]. 

### 2.3. Evidence from In Vitro Studies

Several studies using in vitro BBB models showed that HIV-1 [26,46,47] and viral proteins, including gp120 [39,48,49,50,51], Tat [52,53,54,55,56,57,58], and Nef [59,60], induced endothelial injury and BBB dysfunction; they decreased the expression of endothelial TJs occludin, claudin-5, claudin-1, ZO-1 and ZO-2, decreased transendothelial electrical resistance (TEER), and increased endothelial paracellular permeability, leukocyte adhesion, and transendothelial migration. These HIV- and viral protein-induced endothelial injuries and BBB dysfunction also involved an altered endothelial cytoskeleton, increased cytotoxicity, mitochondrial dysfunction and oxidative stress, and increased expression and secretion of inflammatory cytokines and chemokines, vascular adhesion molecules, MMPs, and other extracellular matrix-degrading enzymes [26,39,46,47,51].

## 3. Factors and Mechanisms Involved in HIV-Mediated Endothelial Injury and BBB Dysfunction

### 3.1. HIV and Infected Leukocytes

Several mechanisms have been shown to mediate HIV transport across the BBB and CNS entry, including transport via mannose-6 phosphate receptor-mediated transcytosis [61] and inflammation-mediated diapedesis of infected leukocytes [62,63,64,65,66]. BBB dysfunction and increased CNS infiltration of leukocytes in HIV infection and HAND have been shown to involve dynamin-related protein-1-mediated mitochondrial dysfunction and oxidative injury [67], inflammation, and downregulation of ZO-1, ZO-2, claudin-1, claudin-5, and occludin [46,68,69]. These HIV-induced effects on the BBB were mediated by Rho-associated kinases, nuclear factor NF-κB, and signal transducer and transcription (STAT)-1 [26,46,68] pathways. Moreover, they were associated with increased MMP-2 and MMP-9 and the dysregulation of brain endothelial efflux transporters, voltage-gated ion channels, calcium-binding proteins, and cytoskeletal and regulatory proteins [64,69,70,71]. Patients with HAND or HIV encephalitis had increased CSF levels of oxidized proteins that correlated with decreased mitochondrial activity and cognitive impairment [72]. 

In HIV/AIDS, cell–cell communications also impact endothelial properties and function. Interactions between primary human brain microvascular endothelial cells (HBMECs) and HIV-infected macrophages have induced endothelial upregulation and activation of pro-inflammatory cytokines and chemokines, including interleukin (IL)-6, TNF-α-induced proteins, interferon-inducible genes, adhesion molecules such as ICAM-1, and transcription factors associated with NF-κB and STAT-1 pathways [46,69]. HIV-induced IL-6 and downregulation of endothelial TJ proteins was associated with increased activation of STAT-1 and STAT-3 and STAT1-mediated induction of interferon-stimulated response element/interferon-γ-activated sequence promoter activity [26,47]. Other studies using HBMECs and hCMEC/D3 cell lines showed that ECs’ exposure to HIV-infected monocytes decreased endothelial TJ and AJ proteins, and increased MMP-9, IL-1β, TNF-α, chemokine ligand-2 (CCL2), and E-selectin. Furthermore, ECs’ interaction with infected monocytes increased monocytes’ transendothelial migration via peroxisome proliferator-activated receptor (PPAR)-α, PPAR-γ, Rho GTPases, and caveolin-1-mediated activation of extracellular signal-regulated kinase-1/2 and protein kinase-B (AKT) [68,73,74,75,76].

### 3.2. HIV Proteins

#### 3.2.1. HIV gp120

Several in vitro and in vivo studies showed that HIV-1 gp120 induced brain endothelial injury, increased BBB permeability and leukocytes’ transendothelial migration. These gp120-induced BBB dysfunctions were associated with calcium dysregulation, were mediated by the C-C chemokine receptor-5, and involved signaling via myosin light-chain kinase, protein kinase-C, and p38 mitogen-activated protein kinase (MAPK) pathways [48,49,50,77,78]. Exposure of HBMECs to gp120 decreased TJ proteins ZO-1, ZO-2, and occludin, decreased AJ proteins such as junctional adhesion molecule (JAM)-1, increased the transcription, expression, and secretion of pro-inflammatory cytokines and chemokines such as IL-6 and IL-8, increased leukocyte adhesion and transendothelial migration via STAT-1 pathways and crosstalk with phosphatidyl inositol-3 kinase (PI3K) and MAP2K pathways [49,50,51], and may also involve endocannabinoids [79]. Exposure of HBMECs to gp120, Tat, or inflammatory mediators (TNF-α and lipopolysaccharides) enhanced the release of occludin-containing extracellular vesicles via small GTPase ADP-ribosylation factor-6 [80]. Endothelial injury and impaired BBB integrity following exposure of brain ECs and mice to gp120 and Tat involved increased ROS, intracellular accumulation of lipid peroxidation products such as malondialdehyde, and decreased activity of glutathione, glutathione peroxidase, and glutathione reductase. The thiol antioxidant N-acetylcysteine amide abrogated these gp120- and Tat-induced effects [81,82,83]. 

#### 3.2.2. HIV Tat

Exposure of brain ECs to HIV-1 Tat induced cytoskeletal changes, dysregulated efflux transporters such as P-glycoprotein and multidrug resistance protein-1, and decreased TEER and the TJ proteins occludin, claudin-1, claudin-5, and ZO-2 [52,53,54,55,56,57,58]. Tat induced apoptosis in ECs and this was associated with mitochondrial dysfunction, increased ROS, and endoplasmic reticulum stress mediated by phosphorylation of eukaryotic translation initiation factor-2α and activating transcription factor-4 [84]. ECs injury following Tat exposure involved increased activator protein-1 (AP-1), ICAM-1, and E-selectin, and the secretion of plasminogen activator inhibitor-1, cytokines and chemokines such as CCL2 and IL-6. These Tat-induced effects involved signaling via AKT, protein kinase-C and -A pathways [85,86,87,88]. Tat-induced brain endothelial injury and BBB dysfunction was also associated with activation/phosphorylation of VE-cadherin [89,90], Rho/Rho-kinase, NF-κB, AP-1, and vascular endothelial growth factor receptor-2 signaling pathways [55,58,91,92,93], oxidative stress and MMP9-mediated cleavage of occludin, resulting in increased BBB permeability [54,55,56], increased ROS and nitric oxide, and decreased intracellular glutathione via NF-κB, AP-1, and Ras pathways [94,95].

#### 3.2.3. HIV Nef, Viral Protein R (Vpr), and p17

Intracellular and extracellular Nef induced mitochondrial dysfunction, caspase activation, and apoptosis in HBMECs [59]. Nef also decreased ZO-1, reduced TEER, increased BBB permeability, and increased chemokines and cytokines such as IL-12, IL-8, IL-6, CCL5, and IL-17A [60]. HIV-1 Vpr induced apoptosis in HBMECs [96], and the HIV-1 matrix protein p17 cross the BBB in vitro and in mice via transcytosis mediated by the CXC motif chemokine receptor-2 [97].

## 4. Endothelial Injury and BBB Dysfunction in HIV Infection and Substance Abuse

Substance abuse is associated with increased risk of HIV transmission and infection, and PLWH with substance use disorders are more likely to be diagnosed late, have poor adherence to ART, and experience accelerated disease progression, increased morbidity, neuropathological complications, and cognitive impairment [98,99]. Substances of abuse that have been investigated for effects on the brain endothelium in HIV/AIDS include cocaine, methamphetamine (meth), alcohol, nicotine/tobacco, opioids, and cannabinoids. 

### 4.1. Cocaine

Cocaine alters the brain endothelium properties and function, and HIV and viral proteins further exacerbate these effects. Exposure of HBMECs to cocaine induced the breakdown of ZO-1 and JAM-2, decreased TEER, altered the endothelial cytoskeleton, upregulated the adhesion molecules ICAM-1, VCAM-1, platelet/endothelial cell adhesion molecule-1, and endothelial leukocyte adhesion molecule-1, and activated leukocyte cell adhesion molecule (ALCAM). Cocaine also upregulated inflammatory cytokines in ECs and increased endothelial paracellular permeability, leukocytes adhesion, and transendothelial migration [100,101,102,103,104,105]. Administration of cocaine to mice increased ALCAM, monocyte adhesion to brain microvessels, and transmigration into brain tissues [101]. HIV and viral proteins further potentiated cocaine-mediated endothelial injury and dysfunction. Cocaine and Tat synergistically decreased HBMECs TEER, ZO-1, and JAM-2, increased endothelial paracellular permeability, and increased monocytes transendothelial migration [102]. Cocaine increased cytokines and neuroinflammation in the CNS of HIV-1 Tg rats [106]. Compared to HIV+ subjects not using cocaine and seronegative controls, brain tissues of HIV+ subjects who abused cocaine showed significant ALCAM upregulation associated with macrophage accumulation around brain microvessels [101]. These cocaine-mediated BBB injuries and dysfunctions have been shown to involve the CCL2/C-C chemokine receptor-2 axis, sigma receptor and platelet-derived growth factor-β activation, NF-κB, MAPK, and PI3K/AKT pathways [100,101]. 

### 4.2. Meth

Meth, HIV-1, and viral proteins can individually and synergistically impair brain cells’ structure, properties, and functions, including cells of the BBB and neurovascular unit [107,108,109]. In vitro studies using hCMEC/D3 cells [110,111,112] and HBMECs [113] showed that Meth and Tat [110,111] or gp120 [113] decreased TEER, decreased the expression of glucose transporter-1 and -3, decreased the expression of TJ proteins ZO-1, occludin, claudin-3, and claudin-5, decreased the expression of AJ proteins JAM-A and JAM-2, and increased endothelial paracellular permeability [110,111,112,113]. Administration of Meth to HIV Tg rats did not alter the HIV-induced decrease in hippocampus TJs and did not increase BBB permeability or gliosis [114]. However, other in vivo studies showed that Meth and Tat or gp120 synergistically decreased TJs and increased BBB permeability in mice, rats, and treeshrews [81,111,115]. Common mechanisms associated with Meth and Tat- or gp120- induced endothelial injury and dysfunction included increased oxidative stress and lipid peroxidation, with increased ROS and malondialdehyde and decreased intracellular glutathione, glutathione peroxidase, and superoxide dismutase activity. Antioxidants such as N-acetylcysteine amide reduced or abrogated Meth and Tat- or gp120-induced BBB impairment [81,111,112,113,115]. 

### 4.3. Alcohol

Alcohol abuse negatively impacts brain function and can exacerbate HIV-associated CNS injury. Binge exposure of HIV Tg rats to ethanol, as well as in vitro exposure of rats and human brain ECs to ethanol and gp120, altered endothelial barrier properties (increased BBB permeability and decreased claudin-3, occludin, and JAM-2) via mechanisms involving the transient receptor potential melastatin-7 ion channel [116]. In vitro studies of HBMECs exposed to ethanol and HIV-1 Nef, Vpr, Tat, or gp120 proteins showed that ethanol potentiated viral protein-induced ECs apoptosis and TNF-α production [96]. Tat and alcohol individually and synergistically altered the BBB, increased macrophage transendothelial migration, and increased neurotoxicity [117]. However, another study showed that alcohol and gp120 individually increased HBMEC permeability, ROS production, and cytoskeletal remodeling, but found no synergistic effect [118]. 

### 4.4. Tobacco

In vivo and in vitro studies showed that nicotine, cotinine, and tobacco smoke extracts have limited effect on the BBB but their combination with protease inhibitors (saquinavir [SQV] and ritonavir [RTV/r]) or gp120 proteins significantly increased BBB injury and dysfunction. Exposure of rats’ brain ECs to nicotine, SQV, or RTV increased ROS, and combination of nicotine and SQV or SQV/r synergistically increased ROS and decreased ZO-1 [119]. In rats treated with nicotine, cotinine, SQV or SQV/r, individual treatment had no major effect on ZO-1 or BBB permeability, but a combination of nicotine or tobacco smoke extracts with SQV or SQV/r significantly decreased ZO-1 and increased BBB permeability (by 2 to 4-fold). These effects were associated with Notch-4 downregulation [119]. Nicotine, cotinine, and their combination target efflux transporters and dose-dependently increased BBB permeability to ^14^C-sucrose and SQV, and increased SQV accumulation in several brain regions (frontal, parietal, and occipital cortex, hippocampus, thalamus, hypo-thalamus, and caudate/putamen) [119,120]. HBMECs exposed to tobacco smoke extracts or gp120 showed decreased viability, mitochondrial membrane potential, ZO-1, occludin, and TEER, and increased BBB permeability. Combined treatment of HBMECs with tobacco smoke extracts and gp120 resulted in a synergistic decrease in TEER and an increase in paracellular permeability, associated with a 2- to 4-fold decrease in endothelial oxygen consumption, mitochondrial membrane potential, ZO-1, and occludin [121]. These data suggest that tobacco use in HIV infection may be associated with increased risk of BBB oxidative injury and dysfunction. 

### 4.5. Opioids

Opioids abuse and overdoses have dramatically increased in the USA and worldwide, and there is evidence that opioids impact HIV immuno- and neuro-pathogenesis [122,123]. In vitro studies showed that chronic low doses of morphine (MOR) had no major effect on HBMECs [124]. However, evidence from other in vivo and in vitro studies suggests that in the presence of HIV proteins, opioids alter the BBB properties and function. Administration of MOR and Tat to mice increased leukocytes trafficking into the CNS and the combination of Tat and MOR potentiated CCL5 upregulation in the CNS [125]. Tat Tg mice treated with fentanyl [126] or MOR [127,128] showed increased BBB disruption and brain inflammatory markers [126,127,128] and increased BBB leakage and recruitment of circulating macrophages into the striatum, hippocampus parenchyma, and perivascular space [127,128]. However, buprenorphine, a partial opioid agonist, decreased CCL2-mediated adhesion of monocytes to HBMECs and transendothelial migration [129]. These results led to suggestions that buprenorphine can reduce HIV-mediated neuroinflammation in addition to its use for the treatment of opioid addiction [129]. Exposure of brain microvascular ECs to MOR and/or Tat altered the expression of ZO-1, occludin and JAM-2, and decreased TEER, which was associated with intracellular calcium release, activation of myosin light-chain kinase, and increased production of pro-inflammatory cytokines [130]. MOR also increased the accumulation of tenofovir, emtricitabine, and dolutegravir in HBMECs and hCMEC/D3 cells [131]. However, these in vitro findings may not necessarily translate to increased CNS entry of these antiretrovirals in vivo. Although MOR increased BBB leakage in Tat Tg mice, it significantly reduced CNS entry of abacavir and dolutegravir, and increased brain P-glycoprotein levels [127], which suggests that MOR may block CNS entry of antiretrovirals by upregulating BBB efflux transporters. 

### 4.6. Cannabinoids

Cannabinoids have been shown to have anti-inflammatory and analgesic properties, and to have a protective effect on the BBB following brain injury. Cannabinoids increased TEER, decreased endothelial VCAM-1, reduced BBB permeability and ischemia-induced BBB damage [132,133], reduced ischemia-induced IL-6 and lactate dehydrogenase in astrocytes [133], increased endogenous antioxidant via NF-κB pathways, reduced cytokines (including TNF-α, IL-1β, and IL-6) expression, and decreased BBB permeability in mice following traumatic brain injury [134]. Cannabinoid-type 2 receptor (CB2R) is expressed in ECs, glial cells, neurons, and brain macrophages [135], and has been shown to mediate cannabinoid effects in vitro and in vivo. Activation of CB2R decreased inflammatory responses in human macrophages and HBMECs, preserved endothelial AJ and TJ proteins, and decreased BBB permeability [136]. CB2R activation decreased inflammation-induced ICAM-1 and VCAM-1, decreased monocytes’ adhesion to the brain endothelium, and decreased BBB permeability in vivo [137,138]. CB2R agonists increased endothelial AJs and TJs occludin, ZO-1, and claudin-5, restored BBB properties, reduced neuroinflammation and neurodegeneration, and attenuated brain injury in murine models of brain hemorrhage [139,140] and traumatic brain injury [141,142].

Cannabis is widely used among PLWH. Considering the evidence above, that cannabinoids attenuated inflammation and BBB damage following brain injury, it has been suggested that cannabinoids’ anti-inflammatory properties could counter HIV-induced neuroinflammation, BBB damage, and CNS injury [135,143]. CB1R and CB2R agonists decreased gp120-induced synapse loss and neuronal death in vitro [144], decreased gp120-induced downregulation of ZO-1, claudin-5, and JAM-1 in HBMECs, and decreased gp120-induced calcium influx, BBB permeability, and monocyte transmigration in vitro and in vivo [79]. In cross-sectional studies of an observational cohort, analyses of the blood and CSF of HIV-infected adults and seronegative controls, cannabis users (moderate and daily users), and non-users, showed that recent use of cannabis was associated with reduced inflammatory markers in both the CSF and blood [143]. Compared to HIV+ non-cannabis users, frequent and daily cannabis use was associated with lower BBB permeability (lower CSF/serum albumin ratio and CSF levels of soluble urokinase plasminogen activator receptor), lower CSF neurofilament-L [145], and lower CSF CCL2 and interferon gamma-induced protein-10 levels associated with better neurocognitive (learning) performance [146]. Longitudinal studies of older HIV-infected adults who were frequent cannabis users, occasional users, and non-users, showed better global cognitive performance among occasional users compared to HIV+ non-cannabis users. However, recent cannabis use was linked to worse cognition, especially regarding memory function [147]. In autopsy studies of brain tissues from HIV-infected humans on long-term ART, subjects with HAND [148], HIV encephalitis, and CNS comorbidities [149] had increased CB1R and CB2R in several brain regions, in neurons, glial cells, and meningeal and perivascular macrophages [148,149]. Increased CB1R was also associated with worse cognition, including poorer memory function and speed of information processing [148]. Furthermore, although CB2R agonists decreased gp120-induced neuronal injury, they had no effect on Tat-induced synapse loss or neuronal death [144]. Thus, it is not yet clear whether cannabinoids abrogate or exacerbate HIV-induced CNS injury and HAND, or the potential effect of the dosage, potency (e.g., delta-9- tetrahydrocannabinol content), and frequency of cannabis use, and polysubstance use.

## 5. Conclusions

The BBB is central to HAND neuropathogenesis. BBB dysfunction during the early stages of HIV infection enables viral entry into the brain, infection of brain cells, and subsequent inflammation, oxidative injury of CNS cells, neurodegeneration, and HAND. This comprehensive review of current evidence from studies in humans, human autopsy brain samples, animals, and in vitro BBB models shows that there is increased injury of the cerebral endothelium, BBB breakdown, and permeability in both symptomatic and asymptomatic HIV infections. HIV-1 proteins such as gp120, Tat, and Nef also induce BBB injury and dysfunction in vivo (Table 1) and in vitro (Table 2), and commonly abused substances such as cocaine, meth, opioids, and tobacco potentiate HIV- and viral protein-induced BBB injury and dysfunction (Table 1, Table 2 and Table 3).

This review also discussed the molecular mechanisms involved in BBB injury and dysfunction induced by HIV, viral proteins, and substances of abuse (overall findings are summarized in Table 1, Table 2 and Table 3). Synergistic increases in BBB damage with substance abuse and HIV or viral proteins would result in increased risk of CNS infection and HAND in HIV-infected substance abusers. As current evidence suggests that ART does not prevent or abrogate HIV-induced BBB injury, it would be important to determine whether ART alters HIV-induced neuropathology, and if any such CNS ART effects are influenced by antiretrovirals drug classes and CNS penetration.

Current evidence suggests that cannabinoids reduce inflammation and HIV-induced BBB injury. However, human studies show conflicting findings, including recent and frequent/daily cannabis use being associated with improved BBB function and better learning in cross-sectional studies and associated with worse cognition/poor memory function in longitudinal studies. Furthermore, human autopsy studies showed that HAND is associated with increased CB1R and CB2R, and demonstrate that increased CB1R is associated with worse memory function and speed of information processing. Thus, whether cannabinoids are neuroprotective or harmful in HIV CNS pathologies and HAND remains to be established.

## Figures and Tables

**Table 1:** In vivo effects of HIV-1, viral proteins, and substances of abuse on the BBB integrity and function, and associated mechanisms.

	Effects on the BBB Structure and Function	Mechanisms
HIV	Downregulation of claudin-5, occludin, ZO-1, and ZO-2, [30,36,45,62,150]. Increased BBB permeability, and infiltration of leukocytes into the brain [36,45,62,150].	Altered expression of ICAM-1 and VCAM-1 [34,150]. Increased levels of C-C chemokines, IFN-γ, CCL5, MIP-1α, MIP-1β, MCP-3, and IP-10 [35].
HIV gp120	Decreased expression of occludin and ZO-1 [81]. Increased BBB permeability [38,81].	Increased ICAM-1, VCAM-1, substance-P, TIMP-1, TIMP-2, MMP-2, and MMP-9 [38,40]. Increased ROS, MDA and protein carbonyl; decreased GSH and GPx activity [40,81].
HIV Tat	Decreased expression of claudin-5, and ZO-1 [41,54]. Disrupted BBB integrity; increased recruitment of activated, phagocytic, and perivascular macrophages into the CNS [42].	Upregulation of ICAM-1, VCAM-1, and AKT activation [88]; autophagy [41].
HIV Nef	Downregulation of claudin-5 [44]. Induced BBB disruption and increased BBB permeability [43,44].	Upregulated MMP-9 [43] and IL-1β [44].
Cocaine and HIV/viral proteins	Cocaine downregulated ZO-1 [101]. Cocaine and HIV-1 increased monocyte adhesion and transmigration [101].	Cocaine induced PDGF-β [101]. Cocaine and HIV-1 increased ALCAM [101] and cytokines [106].
Meth and HIV/viral proteins	Meth and HIV or Tat induced downregulation of ZO-1, occludin, claudin-5, and JAM-A [111,114,115]. Meth and HIV or Tat increased BBB permeability [111,114].	Meth and HIV increased MMP-9 [114]. Meth and Tat downregulated GLUT-1 and GLUT-3 [115]; increased NF-κB [114]; induced ROS, reduced CAT, GPx, and SOD activity; and increased MDA [111].
Alcohol and HIV/viral proteins	Alcohol altered occludin, claudin-5, and ZO-1, and phosphorylated ZO-1 [151,152]. Alcohol increased BBB permeability and transmigration of immune cells into the brain [151,152].	Alcohol activated MMP-3 and MMP-9 [152]. Alcohol and Tat downregulated TRPM7 [116]; increased ICAM-1, IL-1β, MCP-1, CREB, and NF-κB DNA-binding activity; activated ERK1/2 [153].
Tobacco and gp120/antiretrovirals	Nicotine and SQV or SQV/r decreased ZO-1 and increased BBB permeability [119].	Nicotine and SQV or SQV/r decreased Notch-4 and increased ROS [119].
MOR and Tat	MOR or fentanyl and Tat decreased ZO-1, claudin-5, and increased P-gp [126,128]. MOR or fentanyl and Tat increased BBB disruption and permeability [126,128].	Fentanyl and Tat dysregulated inflammatory cytokines [126].

**Table 2:** In vitro effects of HIV-1, viral proteins, and substances of abuse on the BBB integrity and function, and associated mechanisms.

	Effects on the BBB Structure and Function	Mechanisms
HIV	Downregulation of ZO-1, ZO-2, claudin-1, claudin-5, occludin, and JAM-A [46,64,74]; phosphorylation of occludin and claudin-5 [68]; reduced TEER [68]. Increased BBB permeability, leukocyte adhesion and transmigration [26,46,47,64,68,74].	Increased MMP-2 and MMP-9 [64,73,74]; altered ICAM-1, and E-selectin [26,75]; increased IL-6, IL-8, IL-1β, TNF-α, MCP-1, and IFN-inducible genes; activation of SP-1, AP-1, NF-κB, STAT-1, and STAT-3 [26,46,47,73,75]. Increased ROS and redox proteins (peroxiredoxin, SOD); reduced p-eNOS, p-DRP1, and mitochondrial membrane potential [67,69,73]. Activation of Rac1, RhoA GTPases, Rho-kinase, PI3K, PDK1, ERK1/2, and AKT [47,68,73,76].
HIV gp120	Decreased expression of ZO-1, ZO-2, and occludin [49]. Increased BBB permeability, monocyte adhesion, and transendothelial migration [37,49,51].	Increased IL-6, IL-8, ROS, and MDA; decreased activity of GSH, GPx, GR, and CAT [51,82,83]. Activation of STAT-1, p38 MAPK, MEK, and PI3K pathways [51,77]; increased caspase-3 and cytotoxicity [77].
HIV Tat	Decreased expression of claudin-1, claudin-5, ZO-1, ZO-2, occludin, and TEER; increased P-gp, disruption and phosphorylation of VE-cadherin and β-catenin [41,53,54,55,56,57,58,89,90,92,95]. Increased BBB permeability, leukocyte adhesion and transendothelial migration [52,56,86,87,89,90].	Increased E-selectin, ICAM-1 and VCAM-1 [87,88]; MMP-9 [56]; IL-6, PAI-1, MCP-1, and AP-1 [53,85,86,87,91]. Decreased GSH and increased ROS, ER stress, mitochondrial dysfunction, activated UPR, upregulated NOX-2 and NOX-4 [45,89,91,95]. Induced apoptosis, autophagy, and cytotoxicity [41,45,93,95]. Upregulated VEGFR-2 and activated redox-regulated pathways [92,93]. Increased NF-κB binding activity and activation of NF-κB, ERK1/2, IRAK-1/4, MKK JNK, AP-1, PI3K, AKT, FAK, RhoA, ROCK, Ras, PKA, PKC, and PYK2 pathways [52,53,55,56,58,85,87,89,92,93,95].
HIV Nef	Decreased ZO-1, TEER, and altered BBB permeability [60].	Increased IL-12, IL-8, IL-6, CCL5, and IL-17A [60]. Induced PARP cleavage, upregulated Fas/FasL, activation of caspases and mitochondrial apoptotic pathways [59].
Cocaine and HIV/viral proteins	Cocaine, or cocaine and HIV-1 or Tat, decreased ZO-1, JAM-2, and TEER, and increased stress fiber formation [100,102]. Cocaine or cocaine and HIV-1 or Tat increased BBB permeability, monocyte adhesion and transendothelial migration [101,102,104,105].	Cocaine induced activation of ERK1/2, p38 MAPK, JNK, and PI3K/AKT pathways [101]. Cocaine or cocaine and HIV-1 upregulated ICAM-1, VCAM-1, PECAM-1, ELAM-1, and ALCAM [101,104,105]. Cocaine or cocaine and Tat increased inflammatory cytokines, MCP-1 and its receptor in monocytes; increased secretion of TNF-α and IL-6, and activation of NF-κB [100,101,105].
Meth and HIV/viral proteins	Meth, or Meth and gp120 or Tat, decreased ZO-1, JAM-A, occludin, claudin-5, claudin-3, JAM-2, and TEER; and impaired P-gp function [110,111,112,113,115]. Meth or Meth and gp120 or Tat induced BBB disruption, increased BBB permeability and leukocyte transmigration [111,113].	Meth and Tat reduced GLUT-1 and GLUT-3 [115]. Meth or Meth and Tat increased ROS and MDA, reduced CAT, GPx, and SOD activity [111,112]; decreased viability and induced apoptosis in ECs [111,112,115]. Meth and gp120 activate Rho-A GTPase [113].
Alcohol and HIV/viral proteins	Alcohol, alcohol plus gp120 or Tat, decreased claudin-3, claudin-5, occludin, JAM-1, ZO-1, and induced stress fiber formation [116,117,118]. Alcohol and gp120 or Tat increased BBB permeability and macrophage transmigration [117,118].	Alcohol and gp120 or Tat downregulated TRPM7 [116], increased ECs ROS and NO [117,118]. Ethanol and Nef, Tat, gp120, or Vpr increased ECs LDH, TNF-α, and apoptosis [96].
Tobacco and gp120/antiretrovirals	Nicotine and SQV or SQV/r decreased ZO-1 [119]. Tobacco smoke extracts and gp120 decreased occludin, ZO-1, and TEER [121]. Tobacco and SQV or gp120, or tobacco smoke extracts and gp120, increased BBB permeability [119,121].	Nicotine and SQV or SQV/r increased ROS and downregulated Notch-4 [119]. Tobacco smoke extracts and gp120 increased ROS, NF-κB, disrupted mitochondrial function, decreased NRF-2 and EC viability [121].
MOR and Tat	MOR and/or Tat decreased ZO-1, JAM-2, occludin, TEER, and increased P-gp [130]. MOR and/or Tat increased BBB permeability and leukocytes’ transendothelial migration [130].	MOR and/or Tat activated MLCK, increased pro-inflammatory cytokines, and intracellular calcium release [130].

**Table 3:** Interactions between substances of abuse and HIV, viral proteins, and antiretroviral drugs on the BBB integrity and function.

	Interaction	Effects on the BBB
Cocaine + HIV	Additive	Upregulation of ALCAM [101]
Cocaine + Tat	Synergistic	Decreased ZO-1, JAM-2, and TEER, increased BBB permeability, and monocyte transmigration across BBB [102].
Meth + Tat	Synergistic	Decreased ZO-1, occludin, and JAM-A [110,111,115]. Decreased CAT, SOD, and GPx, induced oxidative stress, and increased TRPM2 [111]. Decreased P-gp [110]; decreased claudin-5, GLUT-1, GLUT-3, and cell viability [115].
Meth + gp120	Synergistic	Decreased ZO-1, JAM-2, claudin-3, claudin-5, and TEER; increased leukocytes’ transmigration across the BBB [113].
Meth + gp120 or Tat	Synergistic	Decreased ZO-1, occludin, glutathione, and GPx, increased ROS, protein carbonyls, lipid peroxidation [81].
Ethanol + gp120	Synergistic	Decreased claudin-3, occludin, ZO-1, JAM-2, and TRPM7 [116].
Ethanol + Tat	Synergistic	Increased macrophage migration across BBB [117].
Nicotine + SQR/r	Additive	Decreased Notch-4, and ZO-1, increased ROS, and BBB disruption [119].
Tobacco smoke extracts + gp120	Synergistic	Decreased ZO-1, occludin, and NRF-2; increased ROS, NF-κB, and BBB permeability; decreased TEER, and cell viability [121].
Morphine + Tat	Synergistic	Reduced TEER; increased JAM-2, P-gp, and migration of non-infected and HIV-1 infected leukocytes across the BBB [130].
Fentanyl + Tat	Additive	Decreased claudin-5 [126].

Abbreviations (Table 1, Table 2 and Table 3): ALCAM: activated leukocyte cell adhesion molecule; AP-1: activator protein-1; BBB: blood–brain barrier; CAT: catalase; CCL5: chemokine ligand-5; CNS: central nervous system; CREB: cAMP response element-binding protein; ECs: endothelial cells; ELAM-1: endothelial leucocyte adhesion molecule-1; ER: endoplasmic reticulum; ERK: extracellular signal regulated kinase; FAK: focal adhesion kinase; gp120: glycoprotein 120; GLUT: glucose transporter; GPx: glutathione peroxidase; GR: glutathione reductase; GSH: glutathione; ICAM-1: intercellular adhesion molecule-1; IFN: interferon; IP-10: IFN-gamma-inducible protein-10; IL: interleukin; IRAK-1/4: IL-1 receptor-associated kinase-1/4; JAM: junctional adhesion molecule; JNK: C-jun N-terminal kinase; LDH: lactate dehydrogenase; MAPK: mitogen-activated protein kinase; MCP-1: monocyte chemoattractant protein-1/CCL2; MCP-3: monocyte chemoattractant protein-3/CCL7; MDA: malondialdehyde; MOR: morphine; MEK: mitogen-activated protein kinase; MIP: macrophage inflammatory protein; MLCK: myosin light chain kinase; MKK: mitogen-activated protein kinase kinase; MMP: matrix metalloproteinase; NO: nitric oxide; NOTCH-4: neurogenic locus notch homolog 4; NOX: NADPH oxidase; NRF2: Nuclear factor erythroid 2-related factor 2; PAI-1: plasminogen activator inhibitor-1; PARP: poly (ADP-ribose) polymerase; P-gp: P-glycoprotein; PDGF-β: platelet-derived growth factor β; PDK1: phosphoinositide-dependent kinase-1; PECAM-1: platelet endothelial cell adhesion molecule-1; PI3K: phosphoinositide 3-kinase; PKA: protein kinase-A; PKC: protein kinase-C; p-eNOS: phosphorylated endothelial nitric oxide synthase; p-DRP1: phosphorylated dynamin-related protein-1; PYK2: Protein tyrosine kinase-2 beta; ROCK: Rho-associated protein kinase; ROS: reactive oxygen species; SOD: superoxide dismutase; SQV/r: saquinavir/ritonavir; SP-1: specificity protein-1 transcription factor; STAT: signal transducer and activator of transcription; Tat: Trans-activator of transcription; TEER: transendothelial electrical resistance; TIMP: tissue inhibitor of metalloproteinase; TNF: tumor necrosis factor; TRPM: transient receptor potential melastin; UPR: unfolded protein response; VCAM-1: vascular cell adhesion molecule-1; VEGFR-2: vascular endothelial growth factor receptor-2; ZO: zonula occludens.

## Abbreviations

AIDS	Acquired immunodeficiency syndrome
ALCAM	Activated leukocyte cell adhesion molecule
AJs	Adherent junctions
AKT	Protein kinase B
AP-1	Activator protein 1
ART	Antiretroviral therapy
BBB	Blood–brain barrier
CB1R	Cannabinoid-type 1 receptor
CB2R	Cannabinoid-type 2 receptor
CCL2:	Chemokine ligand 2
CCL5	Chemokine ligand 5
CNS	Central nervous system
CSF	Cerebrospinal fluid
ECs	Endothelial cells
Gp120	Glycoprotein 120
HAND	HIV-associated neurocognitive disorder
HBMECs	Human brain microvascular endothelial cells
HIV	Human immunodeficiency virus
ICAM-1	Intercellular adhesion molecule 1
IL	Interleukin
JAM	Junctional adhesion molecule
MAPK	Mitogen-activated protein kinase
MMPs	Matrix metalloproteinases
MOR	Morphine
Nef	Negative factor
NF-κB	Nuclear factor kappa B
QAIb	CSF/serum albumin quotient
PLWH	People living with HIV
PI3K	Phosphatidyl inositol 3 kinase
PPARα/γ	Peroxisome proliferator-activated receptor alpha/gamma
ROS	Reactive oxygen species
RTV/r	Ritonavir
SQV	Saquinavir
STAT	Signal transducer and activator of transcription
Tat	Trans-activator of transcription
Tg	Transgenic
TEER	Transendothelial electrical resistance
TJs	Tight junctions
TNF-α	Tumor necrosis factor-alpha
SIV	Simian immunodeficiency virus
VCAM-1	Vascular cell adhesion molecule 1
Vpr	Viral protein R
ZO	Zonula occludens
